# What Kind of Brain Structural Connectivity Remodeling Can Relate to Residual Motor Function After Stroke?

**DOI:** 10.3389/fneur.2019.01111

**Published:** 2019-10-23

**Authors:** Wan-wa Wong, Yuqi Fang, Winnie C. W. Chu, Lin Shi, Kai-yu Tong

**Affiliations:** ^1^Department of Biomedical Engineering, The Chinese University of Hong Kong, Shatin, Hong Kong; ^2^Department of Psychiatry and Biobehavioural Sciences, University of California, Los Angeles, Los Angeles, CA, United States; ^3^Department of Imaging and Interventional Radiology, The Chinese University of Hong Kong, Shatin, Hong Kong; ^4^Brain and Mind Institute, The Chinese University of Hong Kong, Shatin, Hong Kong

**Keywords:** chronic stroke, structural remodeling, fiber tractography, regional fractional anisotropy, connection strength, diffusion tensor imaging

## Abstract

Recent findings showed that brain networks far away from a lesion could be altered to adapt changes after stroke. This study examined 13 chronic stroke patients with moderate to severe motor impairment and 13 age-comparable healthy controls using diffusion tensor imaging to investigate the stroke impact on the reorganization of structural connectivity. Each subject's brain was segmented into 68 cortical and 12 subcortical regions of interest (ROIs), and connectivity measures including fractional anisotropy (FA), regional FA (rFA), connection weight (CW) and connection strength (CS) were adopted to compare two subject groups. Correlations between these measures and clinical scores of motor functions (Action Research Arm Test and Fugl-Meyer Assessment for upper extremity) were done. Network-based statistic (NBS) was conducted to identify the connectivity differences between patients and controls from the perspective of whole-brain network. The results showed that both rFAs and CSs demonstrated significant differences between patients and controls in the ipsilesional sensory-motor areas and subcortical network, and bilateral attention and default mode networks. Significant positive correlations were found between the paretic motor functions and the rFAs/CSs of the contralesional medial orbitofrontal cortex (mOFC) and rostral anterior cingulate cortex (rACC), and remained significant even after removing the effect of the ipsilesional corticospinal tract. Additionally, all the connections linked with the contralesional mOFC and rACC showed significantly higher FA/CW values in the stroke patients compared to the healthy controls from the NBS results. These findings indicated that these contralesional prefrontal areas exhibited stronger connections after stroke and strongly related to the residual motor function of the stroke patients.

## 1. Introduction

Structural remodeling of white matter associated with the ipsilesional and contralesional sensorimotor areas has been demonstrated in both animal models of stroke (1) and stroke patients (2) and is found to be associated with the level of motor recovery or impairment (2–4). A number of studies have demonstrated this motor-related structural change in the corticospinal tract (CST) and suggested that the structural integrity of the CST is a major determinant of motor deficit (5). However, in addition to the brain tissue damage localized at the periphery of the lesion, recent studies pointed out that the brain network far away from the lesion could also be altered (6, 7). This alteration is suggested as a secondary white matter degeneration which appears in remote regions interconnected, directly or indirectly, with the primary damaged area (6). Nevertheless, the effect of this remote alteration on motor control/behavior of stroke patients is still under exploration. More understanding of the entire brain adaptation after a stroke might provide a more comprehensive picture of the interactions between structural connectivity remodeling and post-stroke motor function. Such information may be of value in redefining a potential neural substrate that affects post-stroke motor impairment.

Diffusion tensor imaging (DTI) is a noninvasive magnetic resonance technique that measures the random motion of water molecules in brain tissue. It has been used to demonstrate brain abnormalities in stroke (8). This technique is based on the extraction and characterization of the changes in diffusion anisotropy in brain tissue (9), where the diffusion happens to be unequal in all directions. Combined with fiber tractography, which is used to visualize and quantify the integrity of fiber tracts, it might provide a diverse way to reveal the structural remodeling following a stroke (6).

However, using voxel-based assessment for the post-stroke structural changes might be quite challenging, since measurement of the changes can be highly influenced by the variations in stroke topography among stroke population (6). Moreover, since brain lesions often disrupt the neighboring white matter, it might induce erroneous judgment if fiber tracking is based on seed regions extracted from nonlesioned neuroanatomy (10). Therefore, we applied weighted undirected network analysis, which is an alternative and comprehensive approach of assessing structural connectivity (11), to characterize structural brain networks. It has been used to identify detailed abnormalities of network topologies associated with various brain disorders, such as Alzheimer's disease, schizophrenia and Parkinson's disease (12). In this study, a group of 13 chronic stroke patients were included to study their altered fiber pathways in contrast to 13 age-comparable healthy controls.

The flow of the structural connectivity analysis involves dividing up the brain into cortical and subcortical areas to form the ROIs of the network and measures the connectivity between ROIs to characterize the properties of fiber tracts (13). Two main connectivity measures were included to evaluate the post-stroke structural changes: regional fractional anisotropy (rFA) and connection strength (CS). These measures can be used to localize which fiber tracts are affected by the lesions when compared with healthy controls. Then, we compared the differences between stroke patients and healthy controls in terms of rFA and CS from two perspectives, i.e., ROI-wise and connection-wise comparisons. Furthermore, correlations between the upper-limb motor function, which were assessed by clinical assessment scores (Action Research Arm Test [ARAT] and Fugl-Meyer Assessment for upper extremity [FMA-UE]) and the DTI-derived connectivity measures were evaluated in strokes to examine the remodeling of structural connectivity. The results may provide an opportunity to better understand inter-patient connectional variability and to relate it to differences in individual motor function impairment levels.

## 2. Methods

### 2.1. Subjects

This study included 13 first-ever stroke patients (12 males and 1 female, mean age: 54.08 ± 9.01 years) with time since stroke of more than 8 months. They suffered from moderate-to-severe upper-limb impairment with FMA-UE scores lower than 47 (out of 60) (14) (Table 1). Stroke subjects were excluded if they had history of alcohol or drug abuse or epilepsy, bilateral infarcts, uncontrolled medical problems, serious cognitive deficits, comprehensive aphasia or other MRI contraindications. A total of 13 right-handed healthy controls (6 males and 7 females, mean age: 57.62 ± 3.78 years) without cerebral abnormalities were also included to serve as a reference, and their ages were comparable with the stroke subjects. The data of healthy controls were extracted from the open database of the Institute of Psychology, Chinese Academy of Sciences (15). Chi-square test was used to test the differences in gender-distribution between the two subject groups, and significant difference was found in the gender-distribution between the two groups (*p* = 0.015). All the subjects were fully informed of the study protocol and provided the consent form in accordance with the *Declaration of Helsinki*. Additionally, this study was approved by the *Joint Chinese University of Hong Kong - New Territories East Cluster Clinical Research Ethics Committee*.

**Table 1:** Demographic information of stroke patients.

**No**.	**Time since stroke (years)**	**Age range**	**Sex**	**Lesion side**	**Handed-ness**	**Stroke type**	**Lesion location**	**ARAT**	**FMA-UE**	**FMA-SE**	**FMA-WH**
1	11	55–59	M	R	R	i	Brainstem	28	24	17	7
2	7	55–59	M	R	R	i	Insula, IFG, PUT, RO, TP	14	20	15	5
3	3	50–54	F	L	R	h	Insula, RO, PUT	19	34	22	12
4	11	60–64	M	L	R	i	PLIC, PUT	15	22	17	5
5	1	50–54	M	L	R	i	PUT, CN	15	24	17	7
6	1	65–69	M	R	R	h	Insula, ITG, IOG, PUT	8	13	10	3
7	5	40–44	M	R	R	h	Insula, RO, IFG, STG, PUT, TP	9	15	11	4
8	3	40–44	M	R	R	h	Insula, MTG, STG, PUT, TP, RO	11	17	10	7
9	1	45–49	M	R	R	i	MFG, SFG, precentral, SMAR, SMA	3	19	14	5
10	0.67	45–49	M	R	R	h	ITG, MTG, STG, MOG, angular, SMAR	16	17	13	4
11	8	65–69	M	L	R	h	Insula, PUT, IFG, TP	10	22	19	3
12	1	45–49	M	R	R	h	Insula, PUT	12	34	24	10
13	3	60–64	M	R	R	i	Insula, PUT, RO, IFG	4	16	12	4

*ARAT, Action Research Arm Test; FMA-UE, Fugl-Meyer Assessment for upper extremity; FMA_SE, FMA shoulder and elbow movements; FMA_WH, FMA wrist and hand movements; M, Male; F, Female; R, Right; L, Left; PLIC, posterior limb of internal capsule; IFG, inferior frontal gyrus; MFG, middle frontal gyrus; SFG, superior frontal gyrus; SMA, supplementary motor area; ITG, inferior temporal gyrus; MTG, middle temporal gyrus; STG, superior temporal gyrus; IOG, inferior occipital gyrus; MOG, middle occipital gyrus; PUT, putamen; RO, rolandic operculum; TP, temporal pole; CN, caudate nucleus; SMAR, supramarginal; i, ischemic; h, hemorrhage*.

### 2.2. Clinical Assessments

Each patient was assessed by a blinded assessor on their motor function scores of the paretic upper limb using ARAT and FMA-UE. Subdivision was done for FMA-UE score: FMA shoulder and elbow movements (FMA_SE) and FMA wrist and hand movements (FMA_WH). These clinical assessments are used frequently for body function evaluation in upper-extremity rehabilitation training, and their reliability and validity in assessing stroke motor functions have been proven (16). High inter-rater reliability has been shown for FMA-UE score (intra-class correlation coefficient, ICC = 0.98) (17) and for ARAT score (ICC = 0.98) (18).

### 2.3. Image Acquisition

Our stroke subjects were scanned with a 3T MRI scanner (Achieva TX, Philips Medical System, Best, Netherlands) using an 8-channel head coil. The following imaging datasets were acquired: (1) high-resolution T1-weighted anatomical images were acquired using an ultrafast spoiled gradient-echo pulse (T1-TFE) sequence (TR/TE = 7.5/3.5 ms, flip angle = 8°, 308 slices, voxel size = 0.6 × 1.04 × 1.04*mm*^3^); and (2) diffusion-weighted images were acquired using a diffusion-weighted single-shot spin-echo echo-planar pulse (DWISE) sequence (TR/TE = 3,788/88 ms, flip angle = 90°, 60 slices, voxel size = 1.5 × 1.5 × 2*mm*^3^). The diffusion-weighted images were acquired along 32 different diffusion directions with a *b*-value of 1000 *s*/*mm*^2^ and an additional baseline (*b* = 0) image.

The imaging datasets of healthy controls were extracted from an open database (http://fcon_1000.projects.nitrc.org/indi/CoRR/html/ipcas_8.html) and were acquired with a 3T MRI scanner (Siemens Trio Tim, Erlangen, Germany) with a 12-channel head coil. High-resolution structural images were acquired using a magnetization-prepared rapid gradient echo (MPRAGE) three-dimensional T1-weighted sequence (TR/TE = 2,530/3.39 ms, flip angle = 7°, FOV = 256 mm, slice thickness = 1.33 mm). Diffusion-weighted images were acquired using an EPI sequence (TR/TE = 6,600/104 ms, FOV = 230 mm, resolution = 1.8 × 1.8 × 3*mm*^3^). The diffusion-weighted images were acquired along 64 different diffusion directions with a *b*-value of 1000 *s*/*mm*^2^ and an additional baseline (*b* = 0) image. In addition, all brain images were reconstructed and visually inspected for major artifacts (e.g., motion, ringing, wrap around, and neurological abnormalities) before further image processing.

### 2.4. Diffusion Data Processing and Fiber Tractography

Raw diffusion imaging data were pre-processed with a MATLAB toolbox named PANDA (19), which integrates several established packages, including FMRIB Software Library (FSL), Pipeline System for Octave and Matlab (PSOM), Diffusion Toolkit and MRIcron. The processing steps follow a fully automated pipeline, involving correction for eddy-current effect, brain extraction and parcellation, and calculation of diffusion parameters, e.g., FA, eigenvectors and eigenvalues of the diffusion tensor. Deterministic diffusion fiber tracking was performed using standard fiber assignment by the continuous tracking (FACT) method (20). Tracking stops at predefined thresholds of a diffusion-weighted image and a turning angle of 45° to limit the detection of spurious fibers. The tracks were finally smoothed by a B-spline filter to remove any redundant track points and segments. The imaging datasets of patients with right hemispheric lesions were flipped so that the left hemisphere was the ipsilesional hemisphere, whereas the right hemisphere was the contralesional hemisphere.

### 2.5. Structural Connectivity Mapping

In this study, network analysis with a whole-brain analysis approach, which could avoid manually specifying certain seed regions, was adopted to assess structural connectivity changes. The whole brain was divided into 68 cortical and 12 subcortical ROIs, which covered the entire cortices and subcortical structures of both the left and right hemispheres. Based on the T1-weighted image, white and gray matter segmentation was performed in FreeSurfer (Athinoula A. Martinos Center for Biomedical Imaging, USA) to reconstruct and parcellate brain volume in order to produce outputs consisting of labels corresponding to the white matter, the cortex and the deep gray nuclei (21). The labeled mesh of each individual subject was then aligned with his/her diffusion dataset.

**Figure 1:**
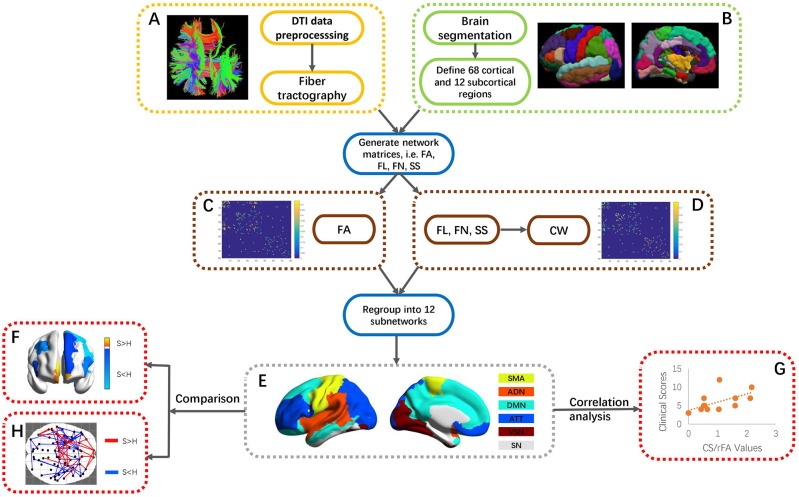
An overall flowchart of this work. **(A)** DTI data preprocessing and reconstruction of fiber tractography. **(B)** Partition into 68 cortical and 12 subcortical ROIs via FreeSurfer segmentation. **(C,D)** Generation of FA and CW matrices through mapping fiber tractography and segmented ROIs. The generation of CW was based on FL, FN, and SS of each ROI. **(E)** Regrouping of 80 ROIs into 12 subnetworks, i.e., SMA, ADN, DMN, ATT, VSN, and SN. **(F)** ROI-wise comparison of CS/rFA values between stroke patients and healthy controls. **(G)** Correlation between CS/rFA values and clinical scores in stroke. **(H)** Connection-wise comparison of CW/FA values between stroke patients and healthy controls using NBS. FA, fractional anisotropy; FL, length of fibers; FN, the number of fibers; SS, surface size of each ROI; SMA, sensory-motor areas; ADN, auditory network; DMN, default mode network; ATT, attention network; VSN, visual recognition network; SN, subcortical network; NBS, network-based statistics.

The outputs of fiber tractography and ROI creation were combined to map connection matrices of FA and CW. Two ROIs were connected if at least one fiber's end-points exist in both ROIs. The overall processing flow for generating network matrices FA and CW is shown in Figures 1A–D. The first matrix, i.e., FA, mapped the average FA along a connection which was linking two ROIs. This measure is commonly used to assess white matter properties in relation to the fiber density, axonal diameter, and myelination status (22). A decline in FA is found to be related to loss of axonal integrity, leading to Wallerian degeneration (23). RFA was calculated as the sum of the FAs for each ROI. The second matrix, i.e., CW, considered the number of fibers, the length of the fibers and the surface size of each ROI. CW is used as a means to capture the connection density between two ROIs (13). A higher value in CW could indicate that the connection has a shorter path length and/or greater number of fibers. The CW matrix was calculated as (13):

(1) $$\begin{array}{r} CW\left(u,v\right)=\frac{2}{Su+Sv}\underset{f\in F\left(u,v\right)}{\sum }\frac{1}{l\left(f\right)} \end{array}$$

where *F*(*u, v*) was the set of fibers connecting ROIs *u* and *v*; *f* was individual fiber within *F*(*u, v*); *S*_*u*_ and *S*_*v*_ were the surface sizes of two ROIs, respectively; *l*(*f*) was the length of fiber *f*; and $\underset{f\in F\left(u,v\right)}{\sum }\frac{1}{l\left(f\right)}$ was sum over all fibers connecting the two ROIs. As shown in Equation (1), CW was composed of the (1) surface size of ROIs, (2) length of fiber, and (3) number of fibers. The surface size was derived from Freesurfer parcellation results, in which the brain was segmented following the Desikan-Killiany Atlas. The surface size of each ROI, and the matrices of fiber length and fiber number from pairs of ROIs were generated by PANDA (19), which was used for diffusion data preprocessing and fiber tracking. The raw CWs were then re-sampled into a Gaussian distribution with a mean of 0.5 and a standard deviation of 0.1 to normalize the scale of the measure for each subject (7). CS was calculated as the sum of all the re-sampled CWs for each ROI, so it can measure the extent to which the ROI was connected to the rest of the network (13). A ROI with higher CS had stronger connections (13), since CS would increase if the connections to the ROI were more intensive.

### 2.6. Statistical Analysis

The whole-brain ROIs were regrouped into 12 subnetworks, including the default mode network, attention network, visual recognition network, auditory network, sensory-motor areas, and subcortical network in both left and right hemispheres, according to the classification method of Tao et al. (24) (Figure 1E). Tao et al. studied the connectivity measures of the regions in the subnetworks with similar functions and dense connections with each other. The subnetworks and their constituting brain ROIs are shown in S_Table 1. A separate two-group between-subject multivariate analysis of variance (MANOVA) was conducted on the CSs and rFAs of the ROIs within each subnetwork to determine whether there were any differences between these two groups. The Box's Test of Equality of Covariance Matrices was conducted a priori to check the assumption of homogeneity of covariance across the groups using *p* < 0.001 as a criterion. Univariate analyses of variance (ANOVAs) were conducted on each dependent measure separately to determine the locus of the statistically significant multivariate effect (Figure 1F). To protect against Type I error, a Bonferroni correction was used to test each ANOVA at the level of 0.05 divided by the number of ANOVAs conducted.

Spearman correlation analysis was used to identify the correlation patterns of structural connectivity properties with motor function (ARAT and FMA clinical scores) (Figure 1G). Moreover, in order to find out whether the correlation patterns would be affected after removing the effects of the CST's structural properties, partial correlation analysis was used to investigate the changes. Partial correlation analysis is often adopted to measure the degree of correlation between two variables regardless of other potential factors' effects. The posterior limb of the internal capsule (PLIC) area is defined as the gap between the thalamus and the lenticular nucleus, and the CST travels through the PLIC in the forebrain before entering the cerebral crus at the base of the midbrain. The PLIC mask was generated based on the segmented T1 images in standard space of the healthy controls. The T1 image in native space of each subject was first aligned with his/her diffusion image and then transformed into the standard space, and matrices for defining the spatial transformations were also generated. The PLIC mask was aligned to the T1 image in native space using reverse normalization based on the warp matrix stored in the header of the T1 image in standard space, and the PLIC mask was now aligned to the diffusion image in native space. Visual checking of the alignment between the PLIC mask and the diffusion image was done for each subject. FA values were averaged within the PLIC mask, and the averaged FA of the ipsilesional PLIC was used as the control variable in the partial correlation analysis. All above statistical procedures were done using IBM Statistical Package for the Social Sciences (SPSS) (version 19). The level of statistical significance was set as *p* < 0.05, and the *p*-value was corrected by false discovery rate (FDR) correction.

Furthermore, network-based statistic (NBS) (25) was used to identify the significant differences of CW and FA matrices between stroke patients and healthy controls from the perspective of the whole brain network (Figure 1H). Large-scale structural connectivity can be modeled as a graph, the ROIs were regarded as nodes of the graph and the connections between the nodes were regarded as edges of the graph. The NBS is a non-parametric statistical method to deal with the multiple comparisons issue on a graph. The method is used to control the family-wise error rate (FWER) when performing mass univariate hypothesis testing on all graph edges. FWER-corrected *p*-values are calculated for each component using permutation testing. The basic premise of permutation testing is that the correspondence between data points and their labels can be randomly rearranged under the null hypothesis without affecting the test statistic. The NBS is a validated method for performing statistical analysis on large networks. A number of studies have used the NBS to identify connections and networks comprising the human connectome that are associated with an experimental effect or a between-group difference. Because the *t*-test in NBS is one-tailed, we performed two-dimensional comparisons between stroke patients and healthy controls, which were “stroke > healthy” and “stroke < healthy” respectively. *T*-test was chosen as the statistical test, and the test statistic threshold was set to 1.8 with the number of permutations specified as 5000 and the FWER corrected significance level specified as 0.05. For each permutation, the steps in the NBS were repeated on the permuted data, involving (1) testing the hypothesis of interest at every connection using the same *t*-test, (2) defining a set of supra-threshold connections using the same threshold and (3) identifying any connected graph components.

## 3. Results

### 3.1. Demographics and Clinical Assessment Scores

**Figure 2:**
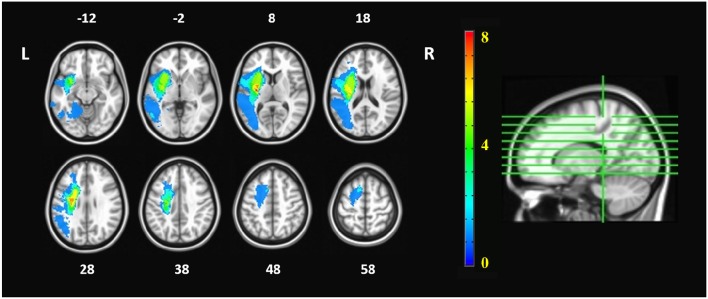
Lesion distribution of stroke patients. The orange numbers in the color bar represented the number of patients who had lesions in the corresponding areas. The white numbers beside the axial images represent the slice number in z coordinate (in mm). The imaging datasets of patients with right hemispheric lesions were flipped, so that the left hemisphere is the ipsilesional hemisphere whereas the right hemisphere is the contralesional hemisphere.

There was no significant age difference between the chronic stroke patients and the healthy controls. The stroke patients had moderate-to-severe upper-limb impairment (ARAT: 12.62 ± 6.54, FMA_SE: 15.46 ± 4.43, FMA_WH: 5.85 ± 2.70), with limited range of motion and functions for the shoulder and elbow joints, and poor wrist and hand functions. More patients had lesions in the right hemisphere (*n* = 9) than in the left hemisphere (*n* = 4), and most of the infarcts were in the territory irrigated by the anterior and middle cerebral arteries. Lesions covered largely the following regions (ordered by number of patients exhibited): putamen (*n* = 10), insula (*n* = 8), rolandic operculum (*n* = 5), inferior frontal gyrus (*n* = 4) and temporal pole (*n* = 4) (Table 1). The lesion distribution of stroke patients was shown in Figure 2.

### 3.2. ROI-Wise Comparison Between Stroke Patients and Healthy Controls

**Figure 3:**
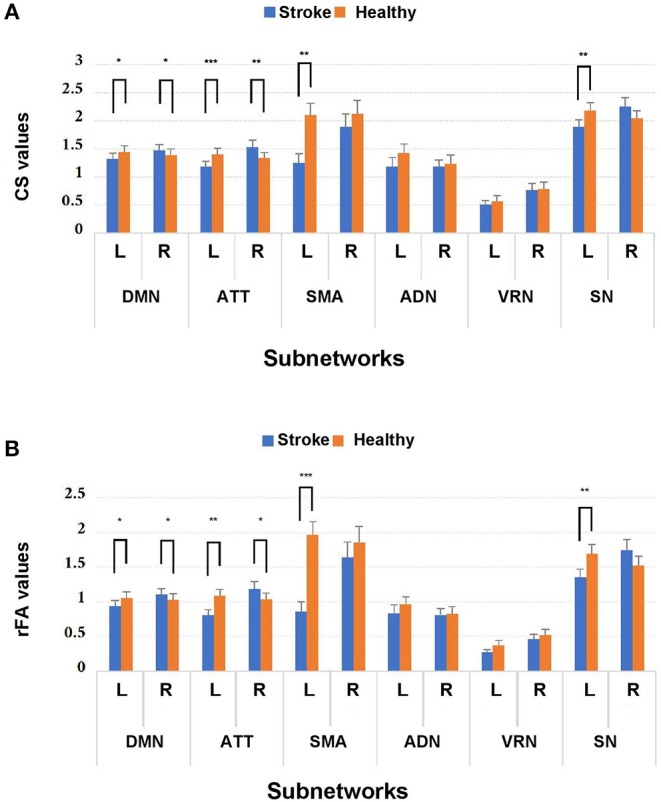
Comparison of **(A)** CS and **(B)** rFA values between stroke patients and healthy controls among the 12 subnetworks. *p < 0.05, **p < 0.01, and ***p < 0.001. Blue and orange bars represent stroke and healthy controls, respectively. DMN, default mode network; ATT, attention network; VRN, visual recognition network; ADN, auditory network; SMA, sensory-motor areas; SN, subcortical network; L, left; R, right.

MANOVA was conducted to determine whether there were any differences between subject groups in the connectivity measures (i.e., CSs and rFAs). From the MANOVA results, significant differences between healthy controls and stroke patients were found in both CSs and rFAs of the regions in the following subnetworks: ipsilesional sensory-motor areas (CS: Wilks' Λ = 0.55, *p* = 0.004; rFA: Wilks' Λ = 0.37, *p* < 0.001), ipsilesional subcortical network (CS: Wilks' Λ = 0.23, *p* = 0.001; rFA: Wilks' Λ = 0.27, *p* = 0.004), ipsilesional attention network (CS: Wilks' Λ = 0.16, *p* < 0.001; rFA: Wilks' Λ = 0.22, *p* = 0.001), contralesional attention network (CS: Wilks' Λ = 0.31, *p* = 0.009; rFA: Wilks' Λ = 0.37, *p* = 0.025), ipsilesional default mode network (CS: Wilks' Λ = 0.31, *p* = 0.018; rFA: Wilks' Λ = 0.36, *p* = 0.04) and contralesional default mode network (CS: Wilks' Λ = 0.36, *p* = 0.044; rFA: Wilks' Λ = 0.32, *p* = 0.02). None of the Box's *M* values were significant (p ≥ 0.001), indicating that there were no significant differences between the covariance matrices. The MANOVA results are shown in Figure 3.

**Figure 4:**
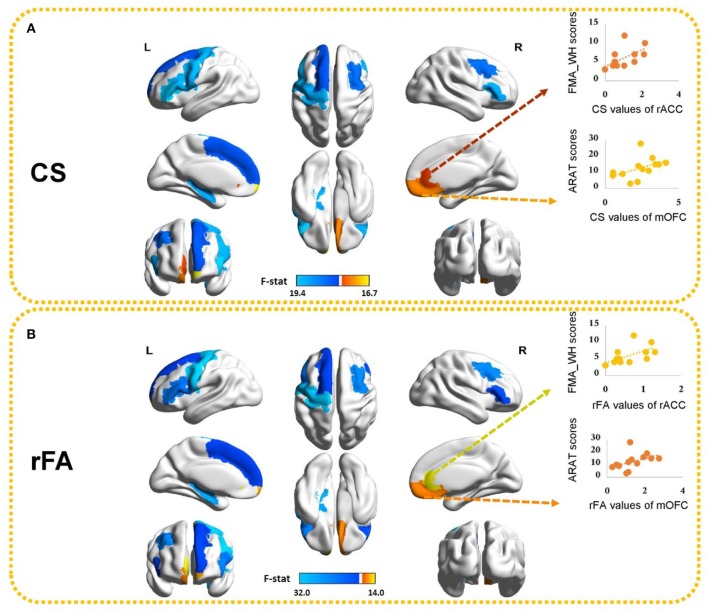
Ten brain ROIs showing significant differences in **(A)** CS and **(B)** rFA between healthy controls and stroke subjects. The values of the color bar correspond to negative logarithm of *p*-values from ANOVA results. Red represents higher CS/rFA values in stroke patients than those in healthy subjects and blue represents lower CS/rFA values in stroke patients than those in healthy subjects. Significant correlations between CS/rFA of the contralesional mOFC/rACC and the clinical scores are also illustrated. mOFC, medial orbitofrontal cortex; rACC, rostral anterior cingulate cortex.

Because MANOVA on some subnetworks showed significant differences between two groups, we further examined the univariate ANOVA results. In the ipsilesional sensory-motor areas, significant univariate group effects on both CS and rFA (*p* < 0.017) were found in the precentral gyrus [CS: *F*_(1, 24)_ = 17.54, *p* < 0.001; rFA: *F*_(1, 24)_ = 31.99, *p* < 0.001]. In the ipsilesional subcortical network, significant univariate group effects on both CS and rFA (*p* < 0.006) were found in the hippocampus [CS: *F*_(1, 24)_ = 16.31, *p* < 0.001; rFA: *F*_(1, 24)_ = 24.21, *p* < 0.001]. In the ipsilesional attention network, significant univariate group effects on both CS and rFA (*p* < 0.006) were found in the pars opercularis [CS: *F*_(1, 24)_ = 17.44, *p* < 0.001; rFA: *F*_(1, 24)_ = 16.68, *p* < 0.001], pars triangularis [CS: *F*_(1, 24)_ = 19.43, *p* < 0.001; rFA: *F*_(1, 24)_ = 18.16, *p* < 0.001] and frontal pole [CS: *F*_(1, 24)_ = 15.85, *p* = 0.001; rFA: *F*_(1, 24)_ = 12.34, *p* = 0.002]. In the contralesional attention network, significant univariate group effects on both CS and rFA (*p* < 0.006] were found in the medial orbitofrontal cortex (mOFC) [CS: *F*_(1, 24)_ = 12.03, *p* = 0.002; rFA: *F*_(1, 24)_ = 11.40, *p* = 0.002] and pars triangularis [CS: *F*_(1, 24)_ = 16.66, *p* < 0.001; rFA: *F*_(1, 24)_ = 11.18, *p* = 0.003]. In the ipsilesional default mode network, significant univariate group effects on both CS and rFA (*p* < 0.005) were found in the superior frontal cortex [CS: *F*_(1, 24)_ = 11.41, *p* = 0.002; rFA: *F*_(1, 24)_ = 10.32, *p* = 0.004]. In the contralesional default mode network, significant univariate group effects on both CS and rFA (*p* < 0.005) were found in the rostral anterior cingulate cortex (rACC) [CS: *F*_(1, 24)_ = 10.27, *p* = 0.004; rFA: *F*_(1, 24)_ = 14.04, *p* = 0.001] and caudal middle frontal cortex [CS: *F*_(1, 24)_ = 12.81, *p* = 0.002; rFA: *F*_(1, 24)_ = 19.6, *p* < 0.001]. The univariate ANOVA results of CS and rFA are shown in Figure 4 and demonstrate the ROIs with significant differences between stroke and healthy controls.

### 3.3. Correlations Between Paretic Upper-Limb Motor Functions and DTI Connectivity

Those ten ROIs, of which the CS and rFA were significantly different between healthy controls and stroke patients, were selected and correlated with the paretic upper-limb motor functions of the stroke patients using Spearman correlation analysis. Significant positive correlations were found between the CS/rFA of the contralesional mOFC and the ARAT scores (CS: rho = 0.729, FDR corrected *p* = 0.05; rFA: rho = 0.729, FDR corrected *p* = 0.05), and between the CS/rFA of the contralesional rACC and the FMA_WH scores (CS: rho = 0.742, FDR corrected *p* = 0.04; rFA: rho = 0.714, FDR corrected *p* = 0.06 [marginally insignificant]). Existing studies have proved that the CST damage correlates with the motor impairment level (26). Therefore, in order to find out the influence on the correlation patterns after removing the effects of the CST's structural properties, we adopted partial correlation analysis. The rFA of the ipsilesional PLIC was revealed to be significantly correlated with the ARAT scores (rho = 0.553, *p* = 0.05), so it was used to represent and quantify the structural integrity of the ipsilesional CST, implying that rFA of the ipsilesional PLIC was the control variable in partial correlation analysis. The CS/rFA of contralesional mOFC and rACC still significantly correlated with the clinical scores (mOFC [CS: *r* = 0.701, *p* = 0.011; rFA: *r* = 0.717, *p* = 0.009]; rACC [CS: *r* = 0.735, *p* = 0.006; rFA: *r* = 0.694, *p* = 0.012]) after removing the effects of CST. This indicated that the correlation between these two areas and the clinical scores was independent of the CST damage. Both CS and rFA of these two ROIs were found larger in stroke patients compared with the healthy controls. The correlation results between CS/rFA values and clinical scores are shown in Figure 4.

The correlations between the clinical scores of motor functions and the connectivity measures of all the 80 regions were also performed and presented in S_Table 2. Significant positive correlations were additionally found between the CS/rFA of the ipsilesional mOFC and the FMA_WH scores (CS: rho = 0.842, uncorrected *p* = 0.000; rFA: rho = 0.781, uncorrected *p* = 0.002). Additionally, the CS of the ipsilesional amygdala and the rFA of the ipsilesional entorhinal cortex also significantly correlated with the FMA_SE and the FMA_WH scores, respectively (uncorrected *p* < 0.01). After the FDR correction, the correlation between the CS of the ipsilesional mOFC and the FMA_WH scores still remained significant (FDR corrected *p* = 0.024).

### 3.4. Connection-Wise Comparison Between Stroke Patients and Healthy Controls

NBS was applied to both CW and FA matrices to identify the connectivity differences between stroke and healthy controls. Both CW and FA showed similar patterns from the results. There were 70 edges with 47 ROIs where the FAs were found to be significantly smaller in stroke compared to the controls (“stroke < healthy”), and there were 37 edges with 32 ROIs where the FAs were found to be significantly larger in stroke compared to the controls (“stroke > healthy”). Similarly, there were 68 edges with 47 ROIs where the CWs were found to be significantly smaller in stroke compared to the controls (“stroke < healthy”), and there were 37 edges with 31 ROIs where the CWs were found to be significantly larger in stroke compared to the controls (“stroke > healthy”). Given the same t-threshold, the number of weakened connectivity was nearly the double of the number of strengthened connectivity, covering widespread areas in both ipsilesional and contralesional hemispheres. The complete information about the connections with the corresponding *t*-test values were shown in S_Table 3. From the NBS results, all the connections linked with the contralesional mOFC and rACC showed significantly higher FA/CW values in the stroke patients compared to the healthy controls (S_Table 2D).

## 4. Discussion

This study aimed to examine the post-stroke structural connectivity reorganization by studying the differences in DTI connectivity measures between stroke patients and healthy controls, and the correlations between the connectivity measures and the paretic upper-limb motor functions of the stroke patients. Altered structural connectivity was found not only in the ipsilesional hemisphere but also in the contralesional hemisphere in chronic stroke patients. Our results showed significant differences between healthy controls and stroke patients in both CSs and rFAs of the ROIs in six subnetworks, including ipsilesional sensory-motor areas, the ipsilesional subcortical network, bilateral attention networks and bilateral default mode networks. From the correlation results, significant positive correlations were found between the paretic upper-limb motor functions and the CS/rFA of the contralesional medial prefrontal cortex (i.e., mOFC and rACC). These correlations remained significant even after removing the effects of the CST's structural properties. Besides, all the connections linked with the contralesional mOFC and rACC showed significantly higher FA/CW values in the stroke patients compared to the healthy controls.

### 4.1. Altered Brain Connectivity in Sensory-Motor Areas

Significant differences between healthy controls and stroke patients were found in both CSs and rFAs of the ROIs in the ipsilesional sensory-motor areas. Sensory-motor areas, including the precentral and postcentral gyri and paracentral lobule, are involved in the control of motor and sensory innervations of the contralateral upper and lower extremities. Among these ROIs, both CS and rFA of the precentral gyrus were found significantly smaller in stroke patients compared with the healthy controls. Our findings are in line with the results of some previous studies. For instance, the study of Wang et al. (27) used graph-theoretical methods to investigate the efficiency of information exchange in the motor areas and found reduced regional centrality in the ipsilesional M1 of the stroke patients. The study of Li et al. (28) assessed the probabilistic fiber tracking of bilateral M1 and found reduced probability of structural connectivity within the pathway connecting the M1 and the contralateral hemisphere in the corpus callosum of the stroke patients compared with the healthy controls.

### 4.2. Altered Brain Connectivity in Subcortical Network

Significant differences were also found in both CSs and rFAs of the ROIs in the ipsilesional subcortical network. For our stroke patients, most of them exhibited their lesions mainly at the subcortical areas (e.g., putamen). It is therefore not surprising to reveal a significant decrease in the connectivity measures of the overall ipsilesional ROIs in the subcortical network due to the direct impact of brain damage induced after stroke. In this network, both CS and rFA of the hippocampus were found significantly smaller in stroke patients compared with the healthy controls. The hippocampus is part of the limbic system, and it facilitates the integration of information from short-term memory to long-term memory and enables the navigation through spatial memory. The major input to the hippocampus originates from the entorhinal cortex through the perforant path. There are many reciprocal connections between the entorhinal cortex and the various cortical and subcortical structures as well as the brainstem. There are also other connections connecting the hippocampus with the cortical and subcortical areas including the prefrontal cortex, the septal nuclei and the hypothalamus. Although there was no infarct located in the hippocampus, the subcortical damage might influence the hippocampal circuitry, leading to the decrease in the connectivity measures of the hippocampus.

### 4.3. Altered Brain Connectivity in Bilateral Attention and Default Mode Networks

Apart from the ipsilesional sensory-motor areas and subcortical network, significant differences were also found in both CSs and rFAs of the ROIs in the bilateral attention and default mode networks, in which reduced CSs and rFAs of the lateral prefrontal cortex and increased CSs and rFAs of the medial prefrontal cortex were found in stroke patients compared with the healthy controls. The attention network and default mode network defined here consist of frontal and parietal areas, which are mainly associated with attention, cognition, planning, motivation, sensory information integration and short-term memory tasks. It has been suggested that there is a close relationship between disparate attentional networks and discrete neural circuitry, and the attentional networks can be affected by specific brain injuries (29). For instance, attention deficits were found associated with post-stroke balance and functional impairment (30), and dysfunctional brain connectivity in the default mode network was also observed after stroke (31).

The connectivity measures of the bilateral lateral prefrontal cortex, including the inferior frontal gyrus, middle frontal gyrus and superior frontal gyrus, were found significantly smaller in stroke patients compared with the healthy controls. Similar findings were also observed in previous study showing a significant decrease in the nodal betweenness centrality of the inferior frontal gyrus, middle frontal gyrus and superior frontal gyrus in the stroke patients compared with the healthy controls (32). The ROI betweenness centrality refers to a fraction of all the shortest paths in the network that involve a certain ROI. The ROIs which are involved in a large number of the shortest paths would have high values of betweenness centrality (33). The observation of this decreased centrality in the prefrontal cortex is in parallel with the atrophy pattern of the frontal lobe after ischemic stroke (34, 35). In our stroke patients, a portion of them exhibited their lesions in these areas in the lateral prefrontal cortex, accounting for the decrease in the connectivity measures of these areas. Reduced connectivity measures were also found in the contralesional lateral prefrontal cortex, which can be supported by the findings from the study of Crofts et al. (6). They revealed reduced communicability, a measure of the ease of transmitting information across a network, in stroke patients not only in the perilesional areas in the ipsilesional hemisphere but also in the homologous areas for a subset of those areas in the contralesional hemisphere. The results may be evidence for secondary degeneration of the structural connectivity interconnecting the remote regions, directly or indirectly, with the primary damaged areas.

On the contrary, the connectivity measures of the bilateral medial prefrontal cortex, including the mOFC, rACC and frontal pole, were found significantly larger in stroke patients compared with the healthy controls. It is quite common to discover reduced connectivity or network measures after stroke compared with the healthy controls due to the destruction of the fiber tracts by the lesions. However, there are also many studies showing increased connectivity or network measures after stroke (2, 6, 32). Increased communicability revealed in the contralesional orbitofrontal cortex has been reported in stroke patients compared to the healthy controls (6). Greater brain activity in the default mode network, including the contralesional anterior cingulate cortex, was also found in stroke patients compared to the healthy controls during resting-state functional MRI (36). These changes could possibly reflect adaptations of white matter structure that responded secondarily to the stroke. Moreover, there is increasing evidence supporting an idea that the stroke-damaged adult brain could attempt to repair itself by producing new neurons even in brain regions where neurogenesis does not normally take place, such as the cerebral cortex (37). Although the knowledge about mechanism regulating the stroke-induced neurogenesis is still incomplete, this potential mechanism for self-repair could demonstrate the possibility of the damaged brain undergoing structural reorganization to compensate the functional loss of primary damaged areas. Interestingly, it is worth mentioning that we also found a dissociative role of the medial and lateral prefrontal cortex in structural reorganization after stroke, revealing decreased connectivity measures in the lateral prefrontal cortex and increased connectivity measures in the medial prefrontal cortex. The prefrontal cortex is recognized to subserve higher executive functions which are involved in task management and planning. The medial and lateral prefrontal cortices have been suggested to pertain to two distinct architectonic trends within the prefrontal cortex (38): the medial prefrontal cortex is in connection with the ventral striatum (consists of the nucleus accumbens and the olfactory tubercle), while the lateral prefrontal cortex is in connection with the dorsolateral striatum (consists of the caudate nucleus and the putamen). Since most of our stroke patients exhibited their lesions mainly at the putamen, the lesions in the putamen might influence the brain connectivity with the lateral prefrontal cortex and be associated with its decreased connectivity measures. Further functional implications in the findings of medial prefrontal cortex are more elaborated in the next section.

### 4.4. Neural Substrates Related to Paretic Motor Functions

Among the 10 ROIs of which the connectivity measures were significantly different between healthy controls and stroke patients, significant positive correlations were found between the paretic upper-limb motor functions and the connectivity measures of the contralesional medial prefrontal cortex, including the mOFC and rACC. These correlations remained significant even after removing the effects of the CST's structural properties. Similar findings have also been reported, showing strengthened functional connectivity among the M1, ventral striatum and other regions, such as the orbitofrontal cortex and anterior cingulate cortex, which belong to the neural circuits for motivation processing, during functional recovery of finger dexterity after spinal cord injury (39). These changes implicate that the neural substrates for motivational regulation of motor learning are involved in functional recovery, suggesting the importance of motivation to functional recovery after damage of the central nervous system such as spinal cord injury and stroke. Further supports can be found from a study revealing that the severity of post-stroke depression is related to the dysfunction of resting-state functional connectivity in the default mode network, including the anterior cingulate cortex where a negative correlation was found between the resting-state functional connectivity index and the severity of anxiety symptoms (40). Moreover, stroke patients with post-stroke depression exhibited reduced gray matter volume and decreased resting-state functional connectivity in the prefrontal cortex, including the orbitofrontal cortex, compared with the patients without post-stroke depression (41). Another study also found a positive relationship between the motor activity level and the FA of the pathway connecting the rACC with the pre-supplementary motor area in people with major depressive disorder (42). Overall, these findings further support the idea that altered structural connectivity in these areas could influence the movement motivation and motor behavior.

In order to further assess connection-wise differences between stroke subjects and healthy controls, NBS was used to identify the differences in structural connectivity between the two subject groups. All the connections linked with the contralesional mOFC and rACC showed significantly higher FA/CW values in the stroke patients compared to the healthy controls. The results were consistent with the results from region-wise comparison and correlation analysis that the connections with these two regions were strengthened after chronic stroke.

### 4.5. Methodological Considerations

Several methodological considerations should be noted while interpreting the results. First, a main limitation in DTI concerns the fiber crossings in the same voxel, so that the real fiber trajectory may not be truly represented by the main diffusion direction derived from the diffusion tensor model which is usually used to estimate a single fiber population within a voxel, and this can in turn lead to erroneous fiber tracking by a nerve fiber tractography algorithm. The fusions, divisions and angulations of the nerve fiber bundles could also induce errors in tractography. Particularly, small fiber tracts and interhemispheric pathways reaching the lateral cortices may be poorly represented due to the complexity of the anatomy in the centrum semiovale and the limited resolution provided by the DTI (13). More advance diffusion imaging methods such as High Angular Resolution Diffusion Imaging (HARDI) and q-ball imaging, and models like multiple tensor or other more complicated fiber models, can be used to enhance the characterization of crossing fibers (43), but at a cost of increased acquisition times. However, our main results focus on large-scale connectivity features that may not be so sensitive to the variation in the small and complex fiber connections.

Second, the voxels of the DTI data in both stroke and healthy groups are not isotropic. It is recommended in much of the literature to use isotropic resolution instead of non-isotropic resolution because non-isotropic voxels, with different in-plane and between-plane resolutions, can cause differential averaging of fiber orientations. This can make the modeling requirements more complicated, leading to model inaccuracy (44). Moreover, FA values measured in regions containing crossing fibers can be underestimated if non-isotropic DTI is used (45). The DTI data acquisition with isotropic voxels is more recommended.

Third, small sample size and heterogeneous subject demographics in this study, including variations in lesion location and volume, time since stroke and stroke type, gender imbalance in stroke group, could limit the generalization of the findings to a larger population and contribute to differences in the connectivity measures and patterns. These findings may not be replicated with different groups of stroke patients with different clinical characteristics. However, based on different analyses, involving MANOVA, correlation and NBS analyses, significant differences and correlations were consistently found in the contralesional rACC and mOFC. The results might implicate the important role of these areas in relating to the structural reorganization and the residual motor functions preserved after chronic stroke.

Fourth, the MRI data of stroke and healthy groups were acquired from different scanners and imaging sequences. The variables introduced by different scanners could devalue the integrity of the results, leading to confusion about whether the results of between-group analysis were owing to the scanner (Philips vs. Siemens) factor or the group (stroke vs. healthy) factor. However, there is an increasing trend toward studies utilizing and pooling the multi-scanner datasets from online databases to advance current research. Studies have been conducted to verify that the scanner differences were substantially less than the group differences, and no significant interaction between scanner and disease was found (46, 47).

Fifth, to investigate the similarity between CS and rFA, bivariate Pearson Correlation was used to check their relationship in both stroke subjects and healthy controls. The results showed that there existed significant correlation between CS and rFA values in both subject groups (stroke subjects: *r* = 0.963, *p* < 0.001; healthy controls: *r* = 0.963, *p* < 0.001]. Although the results derived from these two measures were similar, CS and rFA represent different fiber tract properties. FA is commonly used to measure fiber integrity between two ROIs. The limitation of FA is that it does not consider the surface size of the ROIs, the length of fibers and the number of fibers. CS, on the other hand, considers these parameters, and can measure the extent to which the ROI was connected to the rest of the network. For instance, if only one fiber connects the two ROIs, the FA value can be large as the connection is still intact, but the CW value can be small as the connection density is low. Although the results obtained with rFA and CS were similar, our findings could provide more information about the reorganization of structural connectivity from different perspectives of fiber tract properties.

## 5. Conclusion

In this study, the connectivity measures, both CSs and rFAs, demonstrated similar patterns, showing significant differences between healthy controls and stroke patients with moderate to severe motor impairment in the ipsilesional sensory-motor areas and subcortical network, and bilateral attention networks and default mode networks. Particularly, significant positive correlations were found between the paretic motor functions and the connectivity measures of the contralesional medial prefrontal areas, and the correlations remained significant even after removing the effects of the ipsilesional CST. Further longitudinal studies in larger sample size are recommended to elucidate the role of these involved areas in relation to the residual motor function after stroke.

## Data Availability Statement

The datasets generated for this study are available on request to the corresponding author.

## Ethics Statement

All the subjects were fully informed of the study protocol and provided the consent form in accordance with the Declaration of Helsinki. Besides, this study was approved by the Joint Chinese University of Hong Kong - New Territories East Cluster Clinical Research Ethics Committee (Ref. No. 2015.025).

## Informed Consent

Informed consent was obtained from all individual participants included in the study.

## Author Contributions

WW and YF were responsible for experiments, data analysis, patients' assessment, and paper writing. LS and KT contributed conception and design of the experiment. WC was responsible for experiment arrangement and radiographer contact. All authors contributed to manuscript revision, read and approved the submitted version.

### Conflict of Interest

The authors declare that the research was conducted in the absence of any commercial or financial relationships that could be construed as a potential conflict of interest.
