# Supplementary material for: What Kind of Brain Structural Connectivity Remodeling Can Relate to Residual Motor Function After Stroke?
Source: Front Neurol. 2019 Oct 23;10:1111. doi: 10.3389/fneur.2019.01111 (PMC6819511; doi:10.3389/fneur.2019.01111)
Supplement: Supplementary file 1 [file Table_1.DOCX]

Supplementary Material

**S_Table 1.** The table demonstrated the segmented subnetworks and their constituting brain regions (ROI). Each ROI is symmetric in the ipsilesional and contralesional hemispheres.

| **ROI** | **Subnetwork** |
| --- | --- |
| inferior parietal | Attention Network (ATT) |
| lateral occipital |  |
| medial orbitofrontal |  |
| pars opercularis |  |
| pars orbitalis |  |
| pars triangularis |  |
| rostral middle frontal |  |
| superior parietal |  |
| frontal pole |  |
| bankssts | Auditory Network (ADN) |
| superior temporal |  |
| supramarginal |  |
| transverse temporal |  |
| insula |  |
| middle temporal | Default Mode Network (DMN) |
| precuneus |  |
| superior frontal |  |
| caudal anterior cingulate |  |
| isthmus cingulate |  |
| lateral orbitofrontal |  |
| posterior cingulate |  |
| rostral anterior cingulate |  |
| temporal pole |  |
| caudal middle frontal |  |
| paracentral | Sensory Motor Area (SMA) |
| postcentral |  |
| precentral |  |
| Thalamus | Subcortical Network (SN) |
| Caudate |  |
| Putamen |  |
| Pallidum |  |
| Hippocampus |  |
| Amygdala |  |
| entorhinal |  |
| inferior temporal |  |
| parahippocampal |  |
| cuneus | Visual Recognition Network (VRN) |
| fusiform |  |
| lingual |  |
| pericalcarine |  |
