# Supplementary material for: What Kind of Brain Structural Connectivity Remodeling Can Relate to Residual Motor Function After Stroke?
Source: Front Neurol. 2019 Oct 23;10:1111. doi: 10.3389/fneur.2019.01111 (PMC6819511; doi:10.3389/fneur.2019.01111)
Supplement: Supplementary file 2 [file Table_2.DOCX]

Supplementary Material

**S_Table 2. (A)** T-test values of all the existed connections regarding CW. **(A-1)** S>H: t-test values of connections in stroke group were higher than those in healthy group. **(A-2)** S<H: t-test values of connections in stroke group were smaller than those in healthy group. The threshold of ‘S>H’ and ‘S<H’ in NBS were both set to 1.8. Abbreviations: lh - left hemisphere; rh - right hemisphere.

**(A-1)**

|  | **Connection** | **t-test value** |
| --- | --- | --- |
| **S>H** | ROI_2(lh-caudate)---ROI_4(lh-pallidum) | 5.82 |
|  | ROI_71(rh-rostral anterior cingulate)---ROI_73(rh-superior frontal) | 3.17 |
|  | ROI_59(rh-medial orbitofrontal)---ROI_80(rh-insula) | 3.02 |
|  | ROI_1(lh-thalamus)---ROI_7(rh-thalamus) | 2.96 |
|  | ROI_38(lh-rostral middle frontal)---ROI_43(lh-frontal pole) | 2.9 |
|  | ROI_23(lh-lateral orbitofrontal)---ROI_43(lh-frontal pole) | 2.73 |
|  | ROI_28(lh-paracentral)---ROI_35(lh-precentral) | 2.72 |
|  | ROI_72(rh-rostral middle frontal)---ROI_77(rh-frontal pole) | 2.71 |
|  | ROI_59(rh-medial orbitofrontal)---ROI_77(rh-frontal pole) | 2.7 |
|  | ROI_2(lh-caudate)---ROI_7(rh-thalamus) | 2.41 |
|  | ROI_59(rh-medial orbitofrontal)---ROI_71(rh-rostral anterior cingulate) | 2.33 |
|  | ROI_30(lh-pars orbitalis)---ROI_38(lh-rostral middle frontal) | 2.31 |
|  | ROI_(lh-putamen)3---ROI_23(lh-lateral orbitofrontal) | 2.3 |
|  | ROI_1(lh-thalamus)---ROI_9(rh-putamen) | 2.3 |
|  | ROI_1(lh-thalamus)---ROI_8(rh-caudate) | 2.29 |
|  | ROI_3(lh-putamen)---ROI_6(lh-amygdala) | 2.29 |
|  | ROI_57(rh-lateral orbitofrontal)---ROI_72(rh-rostral middle frontal) | 2.26 |
|  | ROI_3(lh-putamen)---ROI_9(rh-putamen) | 2.26 |
|  | ROI_57(rh-lateral orbitofrontal)---ROI_75(rh-superior temporal) | 2.2 |
|  | ROI_3(lh-putamen)---ROI_44(lh-temporal pole) | 2.13 |
|  | ROI_7(rh-thalamus)---ROI_8(rh-caudate) | 2.04 |
|  | ROI_7(rh-thalamus)---ROI_46(lh-insula) | 2 |
|  | ROI_19(lh-inferior parietal)---ROI_42(lh-supramarginal) | 1.98 |
|  | ROI_19(lh-inferior parietal)---ROI_35(lh-precentral) | 1.89 |
|  | ROI_43(lh-frontal pole)---ROI_77(rh-frontal pole) | 1.89 |
|  | ROI_23(lh-lateral orbitofrontal)---ROI_38(lh-rostral middle frontal) | 1.89 |
|  | ROI_8(rh-caudate)---ROI_9(rh-putamen) | 1.89 |
|  | ROI_1(lh-thalamus)---ROI_26(lh-middle temporal) | 1.88 |
|  | ROI_9(rh-putamen)---ROI_70(rh-precuneus) | 1.88 |
|  | ROI_3(lh-putamen)---ROI_43(lh-frontal pole) | 1.88 |
|  | ROI_39(lh-superior frontal)---ROI_77(rh-frontal pole) | 1.88 |
|  | ROI_25(lh-medial orbitofrontal)---ROI_39(lh-superior frontal) | 1.88 |
|  | ROI_4(lh-pallidum)---ROI_44(lh-temporal pole) | 1.88 |
|  | ROI_3(lh-putamen)---ROI_42(lh-supramarginal) | 1.87 |
|  | ROI_19(lh-inferior parietal)---ROI_13(lh-bankssts) | 1.86 |
|  | ROI_43(lh-frontal pole)---ROI_72(rh-rostral middle frontal) | 1.86 |
|  | ROI_1(lh-thalamus)---ROI_2(lh-caudate) | 1.82 |

**(A-2)**

|  | **Connection** | **t-test value** |
| --- | --- | --- |
| **S<H** | ROI_78(rh-temporal pole)---ROI_80(rh-insula) | 9.38 |
|  | ROI_44(lh-temporal pole)---ROI_46(lh-insula) | 6.12 |
|  | ROI_39(lh-superior frontal)---ROI_73(rh-superior frontal) | 5.2 |
|  | ROI_1(lh-thalamus)---ROI_35(lh-precentral) | 5.08 |
|  | ROI_1(lh-thalamus)---ROI_39(lh-superior frontal) | 4.69 |
|  | ROI_65(rh-pars triangularis)---ROI_72(rh-rostral middle frontal) | 4.35 |
|  | ROI_28(lh-paracentral)---ROI_62(rh-paracentral) | 4.32 |
|  | ROI_(lh-thalamus)1---ROI_33(lh-postcentral) | 4.15 |
|  | ROI_7(rh-thalamus)---ROI_73(rh-superior frontal) | 4.11 |
|  | ROI_(lh-pallidum)4---ROI_33(lh-postcentral) | 4.06 |
|  | ROI_1(lh-thalamus)---ROI_5(lh-hippocampus) | 3.86 |
|  | ROI_29(lh-pars opercularis)---ROI_39(lh-superior frontal) | 3.85 |
|  | ROI_3(lh-putamen)---ROI_33(lh-postcentral) | 3.85 |
|  | ROI_3(lh-putamen)---ROI_35(lh-precentral) | 3.83 |
|  | ROI_41(lh-superior temporal)---ROI_46(lh-insula) | 3.74 |
|  | ROI_49(rh-caudal middle frontal)---ROI_63(rh-pars opercularis) | 3.72 |
|  | ROI_17(lh-entorhinal)---ROI_46(lh-insula) | 3.7 |
|  | ROI_4(lh-pallidum)---ROI_35(lh-precentral) | 3.49 |
|  | ROI_35(lh-precentral)---ROI_39(lh-superior frontal) | 3.37 |
|  | ROI_4(lh-pallidum)---ROI_39(lh-superior frontal) | 3.27 |
|  | ROI_35(lh-precentral)---ROI_69(rh-precentral) | 3.24 |
|  | ROI_54(rh-inferior temporal)---ROI_78(rh-temporal pole) | 3.24 |
|  | ROI_3(lh-putamen)---ROI_30(lh-pars orbitalis) | 3.17 |
|  | ROI_15(lh-caudal middle frontal)---ROI_39(lh-superior frontal) | 3.13 |
|  | ROI_65(rh-pars triangularis)---ROI_73(rh-superior frontal) | 3.12 |
|  | ROI_1(lh-thalamus)---ROI_31(lh-pars triangularis) | 3.07 |
|  | ROI_4(lh-pallidum)---ROI_40(lh-superior parietal) | 2.82 |
|  | ROI_3(lh-putamen)---ROI_31(lh-pars triangularis) | 2.78 |
|  | ROI_53(rh-inferior parietal)---ROI_69(rh-precentral) | 2.74 |
|  | ROI_5(lh-hippocampus)---ROI_27(lh-parahippocampal) | 2.72 |
|  | ROI_28(lh-paracentral)---ROI_69(rh-precentral) | 2.71 |
|  | ROI_3(lh-putamen)---ROI_19(lh-inferior parietal) | 2.7 |
|  | ROI_31(lh-pars triangularis)---ROI_38(lh-rostral middle frontal) | 2.62 |
|  | ROI_15(lh-caudal middle frontal)---ROI_29(lh-pars opercularis) | 2.62 |
|  | ROI_63(rh-pars opercularis)---ROI_73(rh-superior frontal) | 2.48 |
|  | ROI_29(lh-pars opercularis)---ROI_31(lh-pars triangularis) | 2.35 |
|  | ROI_9(rh-putamen)---ROI_57(rh-lateral orbitofrontal) | 2.31 |
|  | ROI_22(lh-lateral occipital)---ROI_41(lh-superior temporal) | 2.3 |
|  | ROI_22(lh-lateral occipital)---ROI_24(lh-lingual) | 2.3 |
|  | ROI_4(lh-pallidum)---ROI_5(lh-hippocampus) | 2.3 |
|  | ROI_1(lh-thalamus)---ROI_20(lh-inferior temporal) | 2.3 |
|  | ROI_4(lh-pallidum)---ROI_30(lh-pars orbitalis) | 2.29 |
|  | ROI_58(rh-lingual)---ROI_80(rh-insula) | 2.28 |
|  | ROI_9(rh-putamen)---ROI_65(rh-pars triangularis) | 2.2 |
|  | ROI_15(lh-caudal middle frontal)---ROI_35(lh-precentral) | 2.17 |
|  | ROI_5(lh-hippocampus)---ROI_17(lh-entorhinal) | 2.15 |
|  | ROI_9(rh-putamen)---ROI_64(rh-pars orbitalis) | 2.11 |
|  | ROI_3(lh-putamen)---ROI_39(lh-superior frontal) | 2.11 |
|  | ROI_1(lh-thalamus)---ROI_4(lh-pallidum) | 2.1 |
|  | ROI_16(lh-cuneus)---ROI_40(lh-superior parietal) | 2.03 |
|  | ROI_28(lh-paracentral)---ROI_73(rh-superior frontal) | 2.03 |
|  | ROI_26(lh-middle temporal)---ROI_41(lh-superior temporal) | 1.96 |
|  | ROI_49(rh-caudal middle frontal)---ROI_69(rh-precentral) | 1.93 |
|  | ROI_54(rh-inferior temporal)---ROI_80(rh-insula) | 1.9 |
|  | ROI_6(lh-amygdala)---ROI_20(lh-inferior temporal) | 1.9 |
|  | ROI_66(rh-pericalcarine)---ROI_80(rh-insula) | 1.89 |
|  | ROI_(rh-thalamus)7---ROI_54(rh-inferior temporal) | 1.89 |
|  | ROI_9(rh-putamen)---ROI_47(rh-bankssts) | 1.89 |
|  | ROI_60(rh-middle temporal)---ROI_80(rh-insula) | 1.89 |
|  | ROI_31(lh-pars triangularis)---ROI_39(lh-superior frontal) | 1.89 |
|  | ROI_22(lh-lateral occipital)---ROI_56(rh-lateral occipital) | 1.88 |
|  | ROI_12(rh-amygdala)---ROI_54(rh-inferior temporal) | 1.88 |
|  | ROI_26(lh-middle temporal)---ROI_29(lh-pars opercularis) | 1.88 |
|  | ROI_4(lh-pallidum)---ROI_31(lh-pars triangularis) | 1.88 |
|  | ROI_17(lh-entorhinal)---ROI_44(lh-temporal pole) | 1.88 |
|  | ROI_64(rh-pars orbitalis)---ROI_65(rh-pars triangularis) | 1.86 |
|  | ROI_1(lh-thalamus)---ROI_30(lh-pars orbitalis) | 1.85 |
|  | ROI_26(lh-middle temporal)---ROI_44(lh-temporal pole) | 1.81 |

**S_Table 2. (B)** T-test values of all the existed connections regarding FA. **(B-1)** S>H: t-test values of connections in stroke group were higher than those in healthy group. **(B-2)** S<H: t-test values of connections in stroke group were smaller than those in healthy group. The threshold of ‘S>H’ and ‘S<H’ in NBS were both set to 1.8. Abbreviations: lh - left hemisphere; rh - right hemisphere.

**(B-1)**

|  | **Connection** | **t-test value** |
| --- | --- | --- |
| **S>H** | ROI_2(lh-caudate)---ROI_4(lh-pallidum) | 5.95 |
|  | ROI_71(rh-rostral anterior cingulate)---ROI_73(rh-superior frontal) | 3.17 |
|  | ROI_59(rh-medial orbitofrontal)---ROI_80(rh-insula) | 2.87 |
|  | ROI_38(lh-rostral middle frontal)---ROI_43(lh-frontal pole) | 2.77 |
|  | ROI_59(rh-medial orbitofrontal)---ROI_71(rh-rostral anterior cingulate) | 2.75 |
|  | ROI_28(lh-paracentral)---ROI_35(lh-precentral) | 2.67 |
|  | ROI_59(rh-medial orbitofrontal)---ROI_77(rh-frontal pole) | 2.65 |
|  | ROI_23(lh-lateral orbitofrontal)---ROI_43(lh-frontal pole) | 2.61 |
|  | ROI_72(rh-rostral middle frontal)---ROI_77(rh-frontal pole) | 2.49 |
|  | ROI_57(rh-lateral orbitofrontal)---ROI_72(rh-rostral middle frontal) | 2.44 |
|  | ROI_19(lh-inferior parietal)---ROI_42(lh-supramarginal) | 2.41 |
|  | ROI_2(lh-caudate)---ROI_7(rh-thalamus) | 2.39 |
|  | ROI_7(rh-thalamus)---ROI_8(rh-caudate) | 2.37 |
|  | ROI_1(lh-thalamus)---ROI_7(rh-thalamus) | 2.35 |
|  | ROI_57(rh-lateral orbitofrontal)---ROI_75(rh-superior temporal) | 2.35 |
|  | ROI_3(lh-putamen)---ROI_6(lh-amygdala) | 2.3 |
|  | ROI_1(lh-thalamus)---ROI_9(rh-putamen) | 2.3 |
|  | ROI_7(rh-thalamus)---ROI_46(lh-insula) | 2.3 |
|  | ROI_3(lh-putamen)---ROI_9(rh-putamen) | 2.29 |
|  | ROI_3(lh-putamen)---ROI_23(lh-lateral orbitofrontal) | 2.29 |
|  | ROI_1(lh-thalamus)---ROI_8(rh-caudate) | 2.27 |
|  | ROI_30(lh-pars orbitalis)---ROI_38(lh-rostral middle frontal) | 2.1 |
|  | ROI_25(lh-medial orbitofrontal)---ROI_37(lh-rostral anterior cingulate) | 2.07 |
|  | ROI_8(rh-caudate)---ROI_9(rh-putamen) | 2.02 |
|  | ROI_13(lh-bankssts)---ROI_19(lh-inferior parietal) | 1.9 |
|  | ROI_43(lh-frontal pole)---ROI_77(rh-frontal pole) | 1.9 |
|  | ROI_1(lh-thalamus)---ROI_26(lh-middle temporal) | 1.89 |
|  | ROI_19(lh-inferior parietal)---ROI_35(lh-precentral) | 1.89 |
|  | ROI_25(lh-medial orbitofrontal)---ROI_39(lh-superior frontal) | 1.89 |
|  | ROI_3(lh-putamen)---ROI_42(lh-supramarginal) | 1.89 |
|  | ROI_9(rh-putamen)---ROI_70(rh-precuneus) | 1.89 |
|  | ROI_39(lh-superior frontal)---ROI_77(rh-frontal pole) | 1.89 |
|  | ROI_3(lh-putamen)---ROI_43(lh-frontal pole) | 1.88 |
|  | ROI_43(lh-frontal pole)---ROI_72(rh-rostral middle frontal) | 1.88 |
|  | ROI_4(lh-pallidum)---ROI_44(lh-temporal pole) | 1.87 |
|  | ROI_1(lh-thalamus)---ROI_2(lh-caudate) | 1.85 |
|  | ROI_3(lh-putamen)---ROI_44(lh-temporal pole) | 1.85 |

**(B-2)**

|  | **Connection** | **t-test value** |
| --- | --- | --- |
| **S<H** | ROI_78(rh-temporal pole)---ROI_80(rh-insula) | 7.66 |
|  | ROI_44(lh-temporal pole)---ROI_46(lh-insula) | 6.31 |
|  | ROI_1(lh-thalamus)---ROI_35(lh-precentral) | 5.31 |
|  | ROI_35(lh-precentral)---ROI_39(lh-superior frontal) | 4.38 |
|  | ROI_28(lh-paracentral)---ROI_62(rh-paracentral) | 4.36 |
|  | ROI_1(lh-thalamus)---ROI_33(lh-postcentral) | 4.35 |
|  | ROI_65(rh-pars triangularis)---ROI_72(rh-rostral middle frontal) | 4.34 |
|  | ROI_4(lh-pallidum)---ROI_35(lh-precentral) | 4.21 |
|  | ROI_1(lh-thalamus)---ROI_39(lh-superior frontal) | 4.19 |
|  | ROI_3(lh-putamen)---ROI_33(lh-postcentral) | 4.17 |
|  | ROI_29(lh-pars opercularis)---ROI_39(lh-superior frontal) | 4.1 |
|  | ROI_1(lh-thalamus)---ROI_5(lh-hippocampus) | 4.08 |
|  | ROI_41(lh-superior temporal)---ROI_46(lh-insula) | 4.08 |
|  | ROI_15(lh-caudal middle frontal)---ROI_35(lh-precentral) | 4.01 |
|  | ROI_4(lh-pallidum)---ROI_33(lh-postcentral) | 3.95 |
|  | ROI_39(lh-superior frontal)---ROI_73(rh-superior frontal) | 3.85 |
|  | ROI_35(lh-precentral)---ROI_69(rh-precentral) | 3.74 |
|  | ROI_3(lh-putamen)---ROI_35(lh-precentral) | 3.7 |
|  | ROI_17(lh-entorhinal)---ROI_46(lh-insula) | 3.69 |
|  | ROI_49(rh-caudal middle frontal)---ROI_63(rh-pars opercularis) | 3.69 |
|  | ROI_4(lh-pallidum)---ROI_39(lh-superior frontal) | 3.29 |
|  | ROI_7(rh-thalamus)---ROI_73(rh-superior frontal) | 3.27 |
|  | ROI_3(lh-putamen)---ROI_4(lh-pallidum) | 3.26 |
|  | ROI_65(rh-pars triangularis)---ROI_73(rh-superior frontal) | 3.2 |
|  | ROI_3(lh-putamen)---ROI_30(lh-pars orbitalis) | 3.19 |
|  | ROI_1(lh-thalamus)---ROI_31(lh-pars triangularis) | 3.18 |
|  | ROI_15(lh-caudal middle frontal)---ROI_39(lh-superior frontal) | 3.05 |
|  | ROI_1(lh-thalamus)---ROI_4(lh-pallidum) | 2.97 |
|  | ROI_54(rh-inferior temporal)---ROI_78(rh-temporal pole) | 2.96 |
|  | ROI_3(lh-putamen)---ROI_31(lh-pars triangularis) | 2.95 |
|  | ROI_4(lh-pallidum)---ROI_40(lh-superior parietal) | 2.83 |
|  | ROI_28(lh-paracentral)---ROI_69(rh-precentral) | 2.74 |
|  | ROI_3(lh-putamen)---ROI_19(lh-inferior parietal) | 2.72 |
|  | ROI_5(lh-hippocampus)---ROI_27(lh-parahippocampal) | 2.71 |
|  | ROI_53(rh-inferior parietal)---ROI_69(rh-precentral) | 2.66 |
|  | ROI_15(lh-caudal middle frontal)---ROI_29(lh-pars opercularis) | 2.61 |
|  | ROI_5(lh-hippocampus)---ROI_17(lh-entorhinal) | 2.48 |
|  | ROI_3(lh-putamen)---ROI_39(lh-superior frontal) | 2.48 |
|  | ROI_29(lh-pars opercularis)---ROI_31(lh-pars triangularis) | 2.43 |
|  | ROI_31(lh-pars triangularis)---ROI_38(lh-rostral middle frontal) | 2.42 |
|  | ROI_9(rh-putamen)---ROI_64(rh-pars orbitalis) | 2.39 |
|  | ROI_63(rh-pars opercularis)---ROI_73(rh-superior frontal) | 2.32 |
|  | ROI_58(rh-lingual)---ROI_80(rh-insula) | 2.31 |
|  | ROI_1(lh-thalamus)---ROI_20(lh-inferior temporal) | 2.3 |
|  | ROI_22(lh-lateral occipital)---ROI_24(lh-lingual) | 2.3 |
|  | ROI_4(lh-pallidum)---ROI_30(lh-pars orbitalis) | 2.3 |
|  | ROI_22(lh-lateral occipital)---ROI_41(lh-superior temporal) | 2.3 |
|  | ROI_4(lh-pallidum)---ROI_5(lh-hippocampus) | 2.27 |
|  | ROI_9(rh-putamen)---ROI_57(rh-lateral orbitofrontal) | 2.27 |
|  | ROI_9(rh-putamen)---ROI_65(rh-pars triangularis) | 2.14 |
|  | ROI_49(rh-caudal middle frontal)---ROI_69(rh-precentral) | 2.13 |
|  | ROI_22(lh-lateral occipital)---ROI_40(lh-superior parietal) | 1.99 |
|  | ROI_28(lh-paracentral)---ROI_73(rh-superior frontal) | 1.98 |
|  | ROI_26(lh-middle temporal)---ROI_44(lh-temporal pole) | 1.95 |
|  | ROI_64(rh-pars orbitalis)---ROI_65(rh-pars triangularis) | 1.95 |
|  | ROI_26(lh-middle temporal)---ROI_29(lh-pars opercularis) | 1.9 |
|  | ROI_4(lh-pallidum)---ROI_31(lh-pars triangularis) | 1.9 |
|  | ROI_16(lh-cuneus)---ROI_40(lh-superior parietal) | 1.9 |
|  | ROI_7(rh-thalamus)---ROI_54(rh-inferior temporal) | 1.9 |
|  | ROI_22(lh-lateral occipital)---ROI_56(rh-lateral occipital) | 1.9 |
|  | ROI_54(rh-inferior temporal)---ROI_80(rh-insula) | 1.9 |
|  | ROI_66(rh-pericalcarine)---ROI_80(rh-insula) | 1.9 |
|  | ROI_6(lh-amygdala)---ROI_20(lh-inferior temporal) | 1.89 |
|  | ROI_1(lh-thalamus)---ROI_30(lh-pars orbitalis) | 1.89 |
|  | ROI_31(lh-pars triangularis)---ROI_39(lh-superior frontal) | 1.89 |
|  | ROI_9(rh-putamen)---ROI_47(rh-bankssts) | 1.89 |
|  | ROI_12(rh-amygdala)---ROI_54(rh-inferior temporal) | 1.89 |
|  | ROI_60(rh-middle temporal)---ROI_80(rh-insula) | 1.89 |
|  | ROI_20(lh-inferior temporal)---ROI_44(lh-temporal pole) | 1.87 |
|  | ROI_49(rh-caudal middle frontal)---ROI_72(rh-rostral middle frontal) | 1.87 |

**S_Table 2. (C)** The NBS results about “stroke > healthy” in FA/CW. All the connections linked with the contralesional mOFC (ROI_59) and rACC (ROI_71) showed significantly higher FA/CW values in the stroke patients compared to the healthy controls.


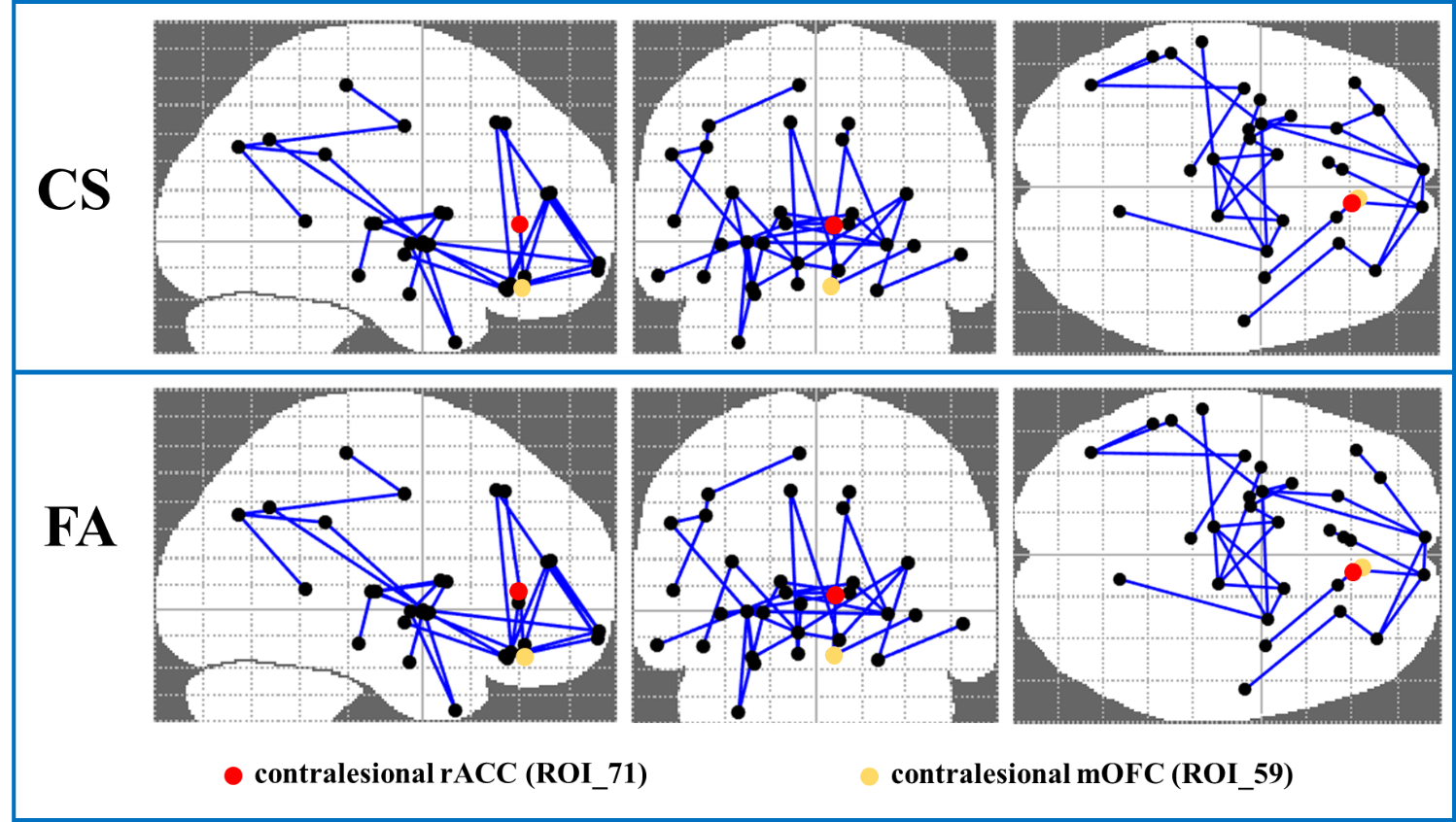


**S_Table 2. (D)** The NBS results about “stroke < healthy” in FA/CW.

**
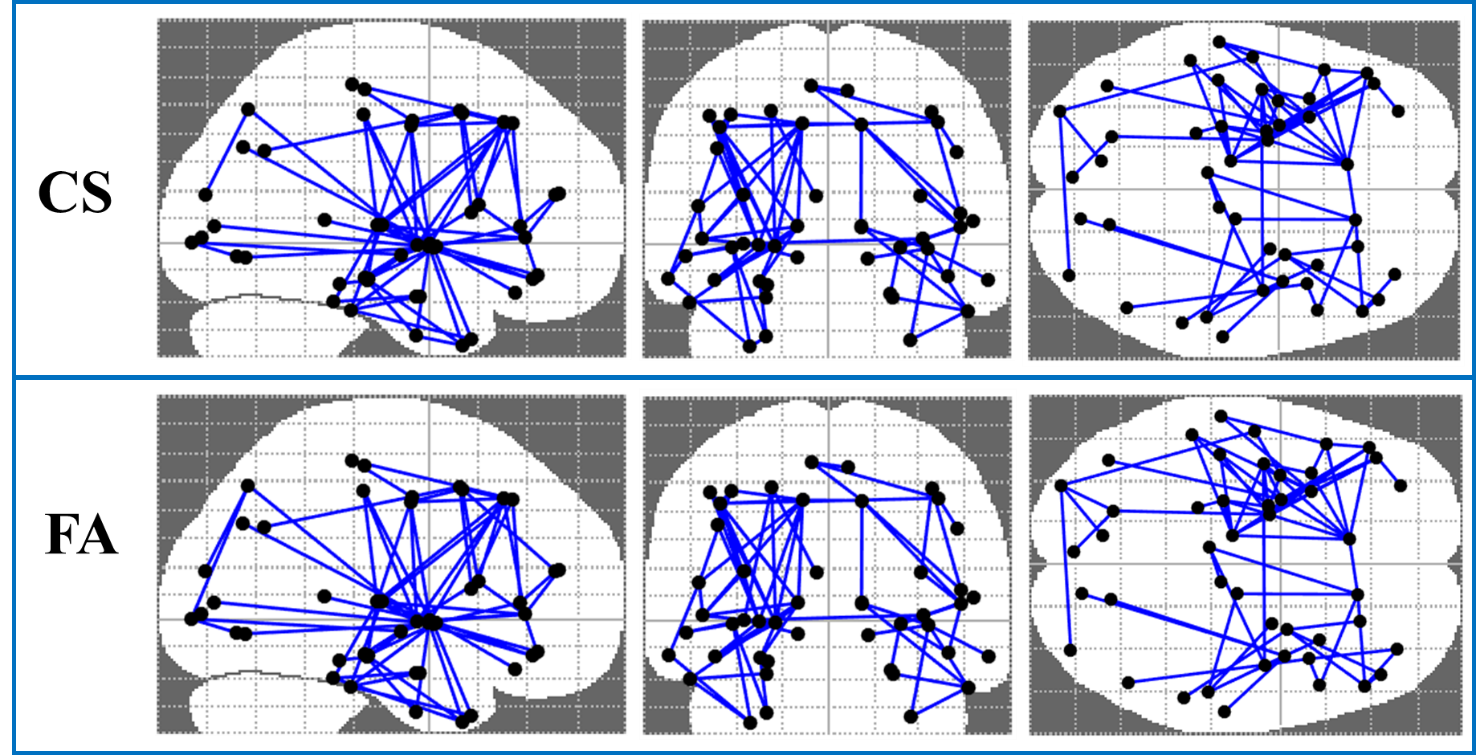
**
